# Tailored Ge-doped fibres for passive electron radiotherapy dosimetry

**DOI:** 10.1371/journal.pone.0235053

**Published:** 2020-07-16

**Authors:** Siti Nurasiah Mat Nawi, S. F. Abdul Sani, M. U. Khandaker, N. M. Ung, K. S. Almugren, F. H. Alkallas, D. A. Bradley

**Affiliations:** 1 Institute for Health Care Development, Sunway University, PJ, Malaysia; 2 Department of Physics, Faculty of Science, University of Malaya, Kuala Lumpur, Malaysia; 3 Clinical Oncology Unit, Faculty of Medicine, University of Malaya, Kuala Lumpur, Malaysia; 4 Department of Physics, Princess Nourah Bint Abdulrahman University, Riyadh, Saudi Arabia; 5 Department of Physics, University of Surrey, Guildford, United Kingdom; Dartmouth College Geisel School of Medicine, UNITED STATES

## Abstract

Study has been made of the thermoluminescence yield of various novel tailor-made silica fibres, 6 and 8 mol % Ge-doped, with four differing outer dimensions, comprised of flat and cylindrical shapes, subjected to electron irradiation. Main thermoluminescence dosimetric characteristics have been investigated, including the glow curve, dose response, energy dependence, minimum detectable dose, effective atomic number, linearity of index and sensitivity of the fibres. The studies have also established the uncertainties involved as well as the stability of response in terms of fading effect, reproducibility and annealing. In addition, dose-rate dependence was accounted for as this has the potential to be a significant factor in radiotherapy applications. The 6 and 8 mol % fibres have been found to provide highly linear dose response within the range 1 to 4 Gy, the smallest size flat fibre, 6 mol% Ge-doped, showing the greatest response by a factor of 1.1 with respect to the highly popular LiF phosphor-based medium TLD100. All of the fibres also showed excellent reproducibility with a standard deviation of < 2% and < 4% for 6 and 8 mol % Ge-doped fibres respectively. For fading evaluation, the smallest 6 mol% Ge-doped dimension flat fibre, i.e., 85 × 270 μm displayed the lowest signal loss within 120 days post-irradiation, at around 26.9% also showing a response superior to that of all of the other fibres. Moreover, all the fibres and TLD-100 chips showed independence with respect to electron irradiation energy and dose-rate. Compared with the 8 mol% Ge-doped optical fibres, the 6 mol% Ge-doped flat optical fibres have been demonstrated to possess more desirable performance features for passive dosimetry, serving as a suitable alternative to TLD-100 for medical irradiation treatment applications.

## 1. Introduction

In delivering a prescribed radiation dose to a well-defined target, the imperative (that of the International Commission on Radiological Units, ICRU) [1] is to achieve accuracy to within < 5% while at the same time minimizing dose to surrounding healthy tissue, a matter discussed in detail by Kry et al (2017) [2]. It has become much more complex to manage the dosimetry of the novel techniques of advancing radiotherapy including small and microbeam therapies [3–4], hypo-fractionation [5]and FLASH radiotherapy [6]. This is due to the various influencing factors which necessarily effect the ability to achieve such accuracy. One particular factor of direct interest to the work herein concerns accurate assessment of the total dose delivered via multiple fractions [7–8]. Such optimized delivery is the product of exhaustive testing, both during acceptance testing as well as in routine use [9].

Thermoluminescence (TL) gives basis to the passive form of dosimetry generally referred to as TLD, commonly used in various popular forms in seeking to validate radiotherapy doses. In present work, application of the more typical phosphor-based commercial forms (eg TLD-100, a medium comprising LiF:Mg, Ti) has been replaced by doped glass media. In earlier work and for various radiation types [10–16] such media have been demonstrated to offer enhanced TL yield, also offering the benefit of being relatively cheap [17–18] as well as being reusable, impervious to liquids, providing potential for in vivo work [19], also the ability for fine fibres fabrication, allowing high spatial resolution work. In passing, it can also be mentioned that access can also be made to other modes of measurement, as in radiation induced attenuation, RIA [20], optically-stimulated luminescence (OSL) and radioluminescence [21–23].

Previous studies of doped glass [9,11,15,17,24–25] have demonstrated TL yield dependencies upon a number of key variables, including concentration of traps (see below), trap stability, light emission via recombination, energy and dose-rate dependence. Control has lead to favourable dosimetric performance, in particular for doped optical fibres, allowing for a wide range of applications [10,15–16,26–27].

High purity silica (eg Suprasil F200, Heraeus, Hanau, Germany) extrinsically doped via a well-disposed impurity can provide the basis of the dominant electron-hole trapping mechanism that gives rise to luminescence, strain generation in fabrication fibre giving rise to one of several subsidiary processes (surface oxidation being another). Optimal doping is particularly important, concentrations beyond the optimum giving rise to self-absorption and with it manifest suppression of the luminescence yield. In production of significant TL Ge has been found to be a particularly effective dopant, providing far greater yields than that of telecommunication fibres doped with Al, Nd, Yb, Er and Sm [12,28–29].

Our first such studies of Ge-doped glass concerned use of commercially available SiO2 (silica) fibres, harnessing the centrally located Ge dopant introduced within the core to provide for the total internal reflection needed for light transport. The Ge significantly increases the number of defect centres within the glass, fortuitous in increasing the TL response [10,15,30–32]. To-date the majority of our studies have made use of commercially available circular cross-section telecommunication fibres, widely available and offering advantageous response in radiotherapy dosimetry applications, comparison being made against competing passive dosimetric systems, TLD or otherwise [10,14–15,26,33].

Building upon the situation analysis provided above, it is to be acknowledged that cylindrical form dosimeters may be less than ideal in catering for the needs of electron dosimetry, a radiotherapy modality making use of superficial dose deposition, as for instance in skin tumour treatments. In present work, investigations have concerned both flat and cylindrical fibres, fabrication leading to altered dose response and sensitivity, potentially also altering factors such as fading. To benchmark performance, comparison has also been made against a well-characterised system, namely TLD-100.

## 2. Materials and method

### 2.1. Sample preparation

Two types (cylindrical and flat) of tailor made Ge-doped optical fibres with dopant concentrations of 6- and 8% were characterised herein for their suitability as TL dosimeters in medical applications. Table 1 shows physical parameters of the cylindrical and flat fibres, obtained during SEM-EDX analysis. Detailed information on the fabrication process has been provided elsewhere [16,27]. Thus said, some additional salient features relevant to the present study are provided herein. A total of 200 fibre dosimeter samples were used herein, each of length of 0.6 ± 0.1 mm, prepared using a commercial optical fibre cleaver (FC-6M, SUMITOMO ELECTRIC Japan). A high precision electronic balance (Metter Toledo) was used to determine the mass of each individual fibre. Note that precise determination of sample mass is important, TL yield being normalized to unit mass to remove the mass dependency. Conventional TLD-100 chips (LiF:Mg, Ti; Thermo Fisher Scientific Inc, Waltham, MA, USA) [34] of dimension 3.2 × 3.2 × 0.89 mm^3^ with a mean mass of 23.6 mg were simultaneously studied to realize the relative response of the fibre dosimeter in practical applications. Dosimetric characterization (i.e. key thermoluminescence properties) of the fibre dosimeter samples and TLD-100 chips was performed following the standard cylic procedures, annealing–irradiation–readout.

**Table 1:** Mean mass, outer cross-sectional dimensions and longest core dimension of the 6 mol% and 8 mol% Ge-doped cylindrical and flat fibres.

Type of Fibre	Ge-dopant concentrations (mol%)	Cross-sectional dimension (μm)	Mass (mg) ± 0.01	Diameter of the core (μm)
**Cylindrical**	**6**	604	3.71	93.60
		483	2.22	71.80
		362	1.27	56.50
		241	0.54	38.40
	**8**	604	3.85	89.00
		483	2.53	73.80
		362	1.37	56.50
		241	0.7	38.40
**Flat**	**6**	85 × 270	0.21	184.00
		100 × 350	0.36	253.00
		165 × 620	1.19	421.00
		200 × 750	1.99	572.00
	**8**	60 × 270	0.18	136.00
		73 × 360	0.31	216.00
		100 × 510	0.59	258.00
		160 × 750	1.39	561.00

### 2.2. Annealing process

Annealing is essential in study of any TL material that is to be investigated as a potential dosimeter. Since present study uses optical fibre samples fabricated locally via the CVD method, the annealing process mitigates against tribo- and chemoluminescence effects, restoring the TL residual signal to essentially that of the background level prior to first use. Prior to annealing, the samples were wrapped in aluminium foil and retained in a ceramic plate to overcome contamination from the multi-user furnace. This arrangement also helps in minimizing the possibility of loss of samples, especially for powdered samples. The fibres were annealed for one hour in a programmable furnace (Micro-controller X; model PXR4/5/7/9) maintained at a temperature of 400 ºC. After annealing, the samples were left in the furnace for 18 hours to slowly cool down to ambient room temperature in order to minimize thermal stress, also resulting in a lesser degree of defect aggregation. The annealed samples were then placed inside a non transparent, light-proof plastic container for subsequent handling.

### 2.3. Samples irradiation

The annealed samples were brought to the Linear Accelerator (LINAC) laboratory located in the Clinical Oncology Unit of the University Malaya Medical Center (UMMC) for exposures to ionizing radiation. A Varian Model 2100C LINAC, one routinely used for radiotherapy, was used for irradiation of the studied samples. A field size of 10 x 10 cm^2^ and a source to skin distance (SSD) of 100 cm was set up as the condition for irradiation of the samples. For evaluation of dose, the irradiation process is carried out by placing the fibres and TLD100 chips in a standard irradiation condition, specifically by placing the TL media centrally within the field of exposure and at the depth of dose maximum, d_max_, measured from the upper surface of a solid water phantom as generally prescribed for such purpose in order to obtain full-scatter conditions. The thickness and dimensions of the solid water phantom is 10 cm and 10 cm x 10 cm respectively. The TLDs were then covered by bolus (tissue equivalent) media in order to provide build up in the beam, delivering the full prescribed dose for the targeted radiation therapy, also increasing the dose at the surface when electron beams are used to treat superficial lesions [35–36]. The fibre dosimeter samples together with the TLD-100 chips were irradiated with electron energies in the range 6–20 MeV, the most commonly used electron energies in the practice of radiotherapy. In the use of a LINAC, the conventional dose rate is expressed in terms of Monitor Units (1 MU = 1 cGy); in this regard, the samples were exposed to dose-rates in the range 100–600 MU min^-1^.

### 2.4. Samples readout

The irradiated samples were removed from the LINAC irradiation chamber and brought to the measurement laboratory located at the Physics Department, University Malaya. A Harshaw 3500 TLD reader supported by WinREMs software was used to perform the readout process of the fibre dosimeters and TLD-100 chips. The readout system mainly consists of a photomultiplier tube (PMT) and an Electrometer, recording the PMT signal as a charge or current. During the readout process the machine is supplied with a slow flow of nitrogen gas in order to reduce surface effects such as oxidation, chemiluminescence and triboluminescence.

In order to acquire the TL signal, the TLD reader was set up with the following parameters; preheat temperature of 50 ºC for 40 s, heating rate cycle or ramp rate of 10 ºCs^-1^, and a maximum temperature of 400 ºC. The aforementioned parameters were optimized to obtain an optimal glow curve, free of the effects of superficial traps. The ramp rate was varied to determine the time–temperature profile (TTP), ensuring complete capture of the TL glow curve under the optimum conditions. The TL readings obtained for each dosimeter were normalized to their respective mass, obtaining results in nC/mg.

## 3. Results and discussion

### 3.1. Annealing temperature effect and annealing time effect

**Fig 1:**
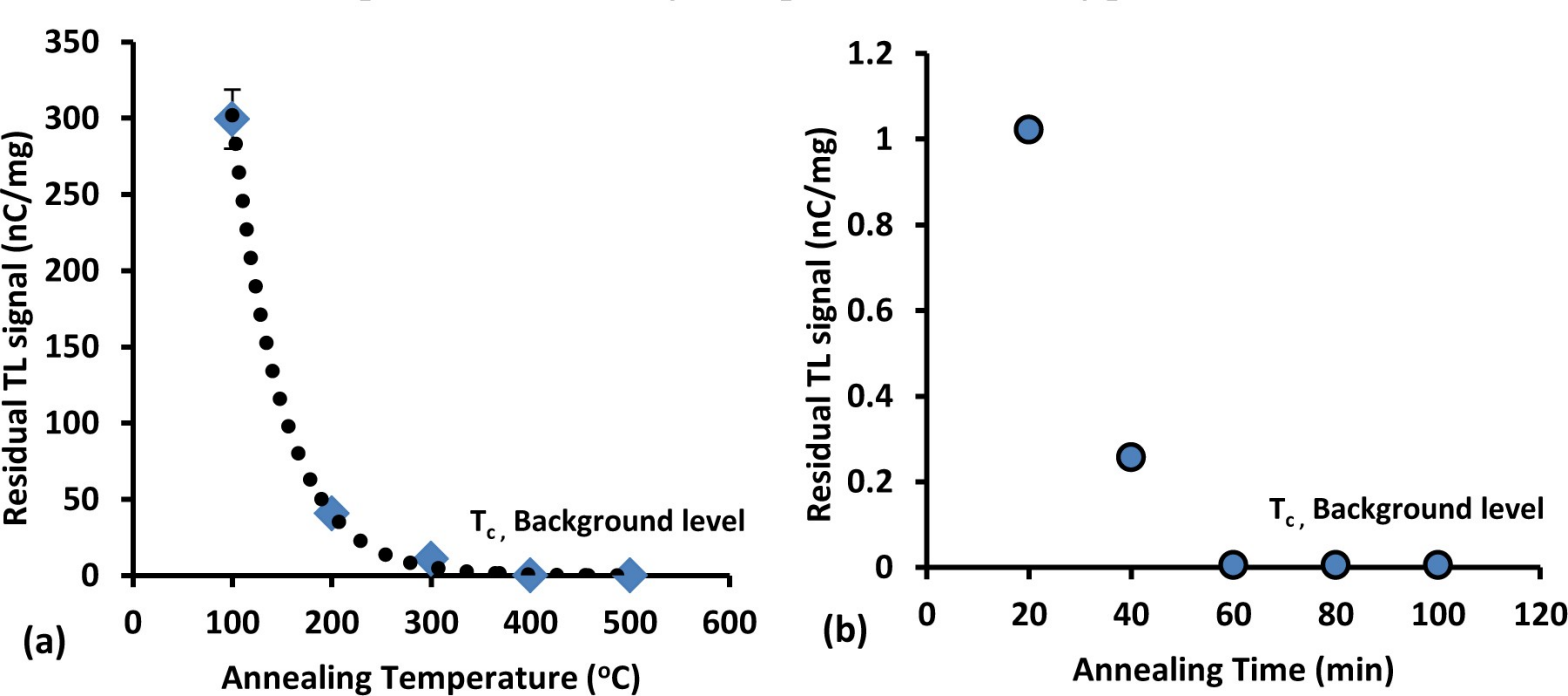
Residual TL signal against (a) annealing temperature and (b) annealing time.

In TL applications, when a new TL material is to be used for the first time, it is necessary to perform an initial annealing investigation. The intent of this is to obtain the highest sensitivity TLD for the lowest potential intrinsic background, finding the best combination of annealing temperature and time to eliminate any effect of previous irradiation, also obtaining the greatest reproducibility for both TL and background. The experiments were performed using different annealing temperatures, from 100 ºC up to 500 ºC in increments of 100 ºC at a constant annealing time of one hour for each temperature. For the optimal annealing time, the experiment was carried up using a fixed temperature of 400 ºC for different annealing times from 20 to 100 minutes, increased in an increment of 20 minutes. A total of 25 fibres were irradiated using a dose of 10 Gy delivered by ^60^Co irradiation at a dose-rate of 0.525 Gy/sec. The best combination of parameters, between temperature and time, were obtained based on the resultant graphs of residual TL signal versus temperature (T) and residual TL signal versus time (t). The most favourable choice is the one in which the TL residual signal is practically the same as background. Fig 1(A) shows the residual TL signal against the annealing temperature. From this graph, it is apparent that the reading of residual TL signal decreases with increase in temperature, remaining constant at the temperature of 400 ^o^C. In other words, after a threshold temperature value T_c_ (equal to 400 ^o^C in the present case) the residual TL signal becomes identical to background, all previous irradiation history being erased from the samples. The graph of residual TL signal versus annealing time is presented in Fig 1(B). The graph shows the values of residual signal to be inversely proportional to the annealing time, remaining constant after 60 minutes. Therefore the optimal time to erase all irradiation memory from the samples is found to be 60 minutes. Based on both graphs, the best combination of temperature and time for annealing condition is 400 ^o^C for one hour. The values obtained are important in defining the optimum annealing procedure.

### 3.2. Minimum detectable dose

The minimum detectable dose (MDD) is important in low dose measurements where MDD is the smallest dose in response that is statically significant different from a background signal. Another way to determine the MDD was proposed by Furetta so-called lower limit is defined as the dose which gives three times the standard deviation of the zero doses reading of the dosimeter [33]. In present research, MDD for all of the fibre samples are calculated as in Eq (1) [37]:

$$D_{o}=\left(Bmean+2\sigma \right)\;F,\;F=1/m$$ (1)

where D_o_ = minimum detectable dose, B_mean_ = TL background signal obtained from the TL samples annealed but then not irradiated, σ = standard deviation of the mean background, F = TL system calibration factor for each type of the optical fibres expressed in GynC^-1^ and m = slope obtained from TL dose response for each TL materials.

The results of minimum detectable dose (MDD) for all of the samples are tabulated in Table 2. In the present work the MDD values show the lowest doses capable of being detected by the fibres to be encouragingly low for electron irradiation. In particular, while the MDD for 6 mol% Ge-doped silica fibres were found to be above 1mGy for all but one type, the exception was for cylindrical fibres of 483 μm external diameter with the MDD below 1mGy. The MDD for 8 mol% Ge-doped fibres shows values lower than 1mGy for all of the flat fibres, while for all but one of the cylindrical fibres the MDD was higher than 1 mGy, the exception being that of outer diameter fibres of 604 μm. Establishing the relationship between TL sensitivity, TL dosimeter material and type of TLD reader is important in obtaining a low detection dose.

**Table 2:** Minimum detectable dose for dimension of 6 and 8 mol% cylindrical and flat Ge-doped fibres.

Type of Fibre	6 mol%		8 mol%	
	Cross-sectional dimension (μm)	MDD (mGy)	Cross-sectional dimension (μm)	MDD (mGy)
**Cylindrical Fibre**	**604**	1.51	**604**	0.58
	**483**	0.78	**483**	1.15
	**362**	1.63	**362**	1.77
	**241**	1.39	**241**	1.28
**Flat Fibre**	**85 × 270**	1.21	**60 × 270**	0.02
	**100 × 350**	1.59	**73 × 360**	0.02
	**165 × 620**	1.11	**100 × 510**	0.06
	**200 × 750**	1.33	**160 × 750**	0.02

### 3.3. Effective atomic number (Z_eff_)

In the present study, the Z_eff_ of all cylindrical and flat 6 and 8 mol % Ge-doped optical fibres were investigated in accord with the Mayneord equation [38]. SEM–EDX analysis was carried out to map the relative distribution of the Ge dopant inside the fibres, also identifying the elemental composition (germanium, oxygen and silicon) (see Table 3). The values of Z_eff_ are found to range from 11.59 to 15.83 for fibres with 6 mol% Ge while it is 13.19 to 16.53 for fibres with 8 mol% Ge. The smallest dimension 6 mol% Ge-doped flat fibre (85 x 270 μm) is associated with the least value, remaining greater than the value for soft tissues (7.42) but within the range of human-bone (11.6–13.8) (see Table 3).

**Table 3:** Weight percentage and Zeff of 6 and 8 mol% Ge-doped optical fibres using EDX analysis.

Type of Fibre	Ge-dopant concentrations (mol%)	Cross-sectional dimension (μm)	Weight percentage (%)			Z*eff*
			O	Si	Ge	
**Cylindrical**	**6**	**604**	53.99	42.18	3.83	14.95
		**483**	52.57	42.45	4.98	14.93
		**362**	53.1	41.67	5.23	15.83
		**241**	53.38	42.49	4.12	15.11
	**8**	**604**	51.49	42.45	6.06	16.03
		**483**	53.59	40.3	6.11	15.33
		**362**	52.79	39.68	7.53	15.61
		**241**	53.86	41.15	4.99	15.47
**Flat**	**6**	**85 × 270**	59.29	40.54	0.17	11.59
		**100 × 350**	56.14	38.12	5.74	13.65
		**165 × 620**	56.13	39.49	4.38	13.87
		**200 × 750**	56.57	39.02	4.41	13.97
	**8**	**60 × 270**	55.78	43.82	4	16.53
		**73 × 360**	56.57	39.02	4.41	13.72
		**100 × 510**	48.99	49.71	1.3	14.8
		**160 × 750**	45.15	45.47	9.38	13.19

### 3.4. Glow curve

**Fig 2:**
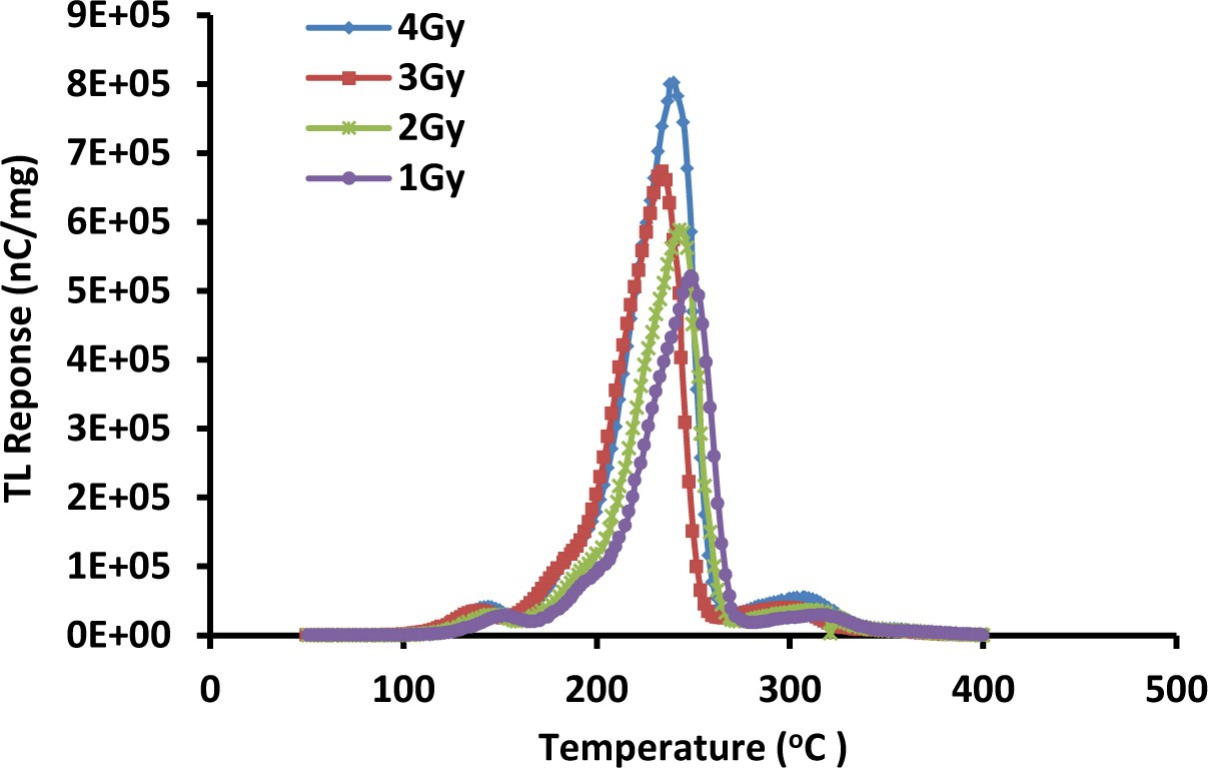
Typical TL glow curve for TLD-100 for delivered doses in the range 1–4 Gy at fixed energy and dose-rate of 6 MeV and 400 MU/min respectively, determining the effect of dose on the TL glow curve.

**Fig 3:**
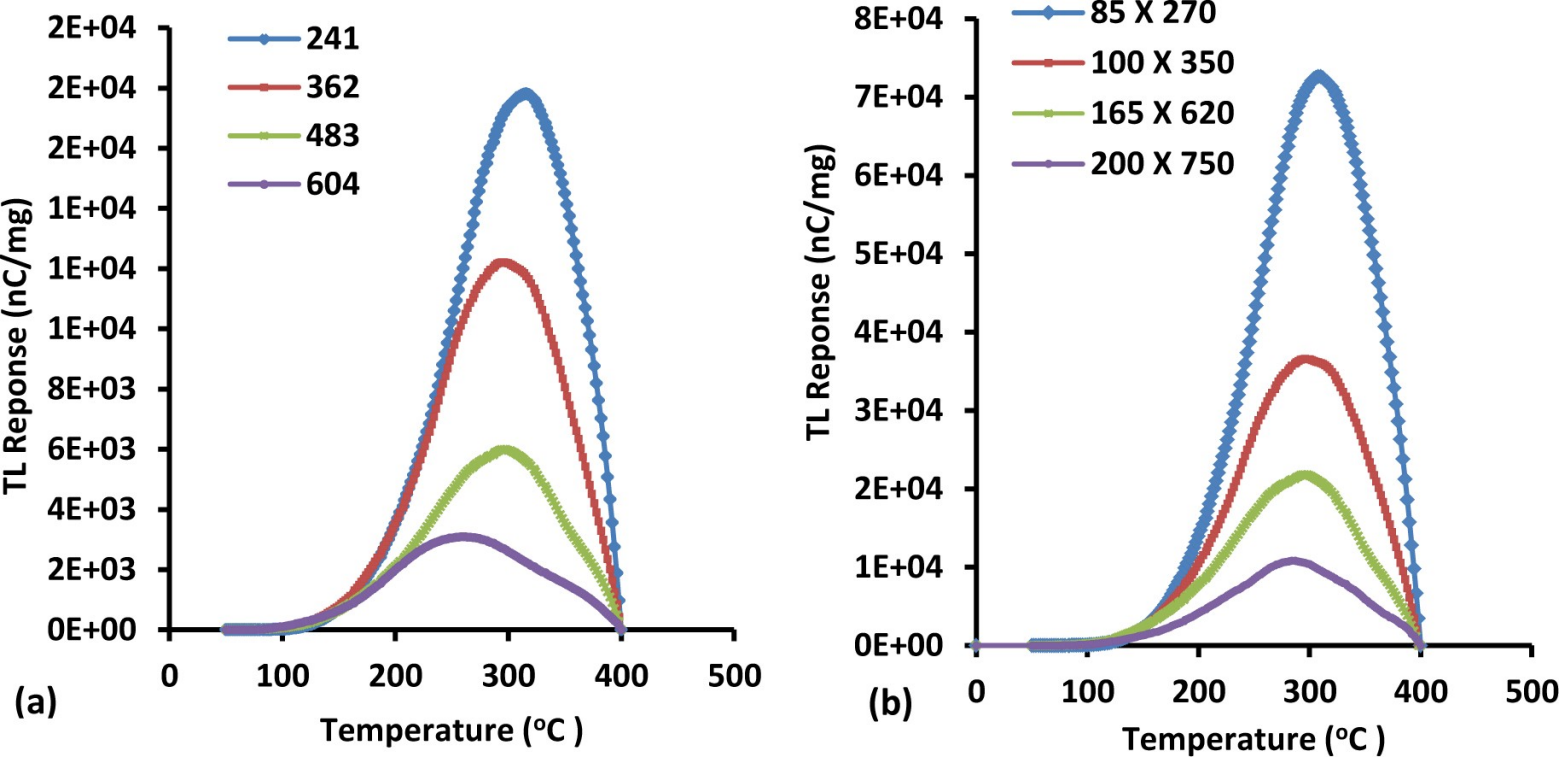
Typical Ge-doped (a) cylindrical and (b) flat fibre TL glow curves, all for a dose of 2 Gy at 6 MeV and 400 MU/min for energy and dose rate respectively, encompassed between temperatures of ~ 50 ^º^C and 400 ^º^C in order to determine the effect of different diameters of fibre on the TL glow curve.

An important physical factor in determining the stability of a given TL material for medical applications is the temperature at which the peak of the glow curve occurs. Most commercially available TL dosimeters have high glow peak temperature values, indicative of deep electron traps to the extent that the stored signal is highly stable for several months to years [39]. Figs 2 and 3(A) and 3(B) show the typical glow curves for the TLD-100 and fibres studied herein, respectively. Based on the figures, the glow curves of TLD-100 and Ge-doped fibre are similar in shape, the general peak structure of the TL glow curves remaining unchanged as a result of repeat cycles of irradiation at various doses. The exception is that the position of the largest peak depends on how much dose is delivered to the TL materials. With increase in the dose delivered, the position of the highest intensity peak in the glow curve also increases. In other words, the position of the highest intensity peak of the glow curve is directly related to amount of dose delivered and the results support findings from previous study [15,39]. This is because with increase in the dose delivered, an increasing number of electrons are excited to the conduction band and commensurately more electrons are trapped at the various electron trap centers. During the thermal stimulation process, these electrons are released from the traps, reflecting the higher TL response with irradiation dose [15]. Therefore, in conclusion, the behaviour displayed by the TL glow curve of the fibres has been seen to be practically the same as TLD-100 (resulting narrower peaked structure within the range 234 ºC– 250 ºC as seen in Fig 2), as found in current research. Both dosimeters show the largest intensity peak of the TL glow curve to be strongly dose dependent. Moreover, the area under each graph shows the number of electrons released from traps. Therefore, the glow peak maximum indicates the maximum in the number of electrons released from traps when association the radiation energy deposited [27].

The glow curves for the various cross-section fibres of 6 mol% is presented in Fig 3(A) and 3(B), this being a display of intensity of luminescence as a function of temperature for a fixed dose, dose-rate and energy of 2 Gy, 400 MUmin^-1^ and 6 MeV respectively. The samples were heated using a fixed linear heating-rate of 10°C/s and the maximum readout temperature was 400 ºC.

For each cylindrical fibre cross-section, a broad peaked structure is observed, the peak maxima occurring at temperatures within the range 276 ºC– 320 ºC while for flat fibres this is shown to be within the range 284 ºC—312 ºC. As a general scrutiny, both of the fibre types show no remarkable change in temperature at which the maximum in the TL glow curve peak occurs. In more detail, the graphs show that the smaller dimension fibres of both types display the greater TL glow curve peak position, exhibiting 18590 nA and 74836 nA of TL current for the smallest diameter cylindrical fibre (241 μm) and smallest cross-sectional dimension flat fibre (85 x 270 μm) respectively after normalization to unit mass of fibre. Conversely, the larger dimension fibres of both types show the lowest TL glow curve peak position, displaying a TL intensity of 3490 nA and 11150 nA for the largest diameter cylindrical fibre (604μm) and largest cross-sectional dimension flat fibre (200 x 750 μm) respectively after normalization to unit mass of fibre.

From the graphs, the shape of the glow curves were observed to remain unchanged for all fibres even though different dimensions of fibre were used while for the largest intensity peak, the position of the TL glow curve did change to a degree dependent on the dimension of fibre used. The patterns obtained are quite similar with the results obtained from the effect of doses on the TL glow curve in which a change in the largest intensity peak position was noted.

### 3.5. Dose response

**Fig 4:**
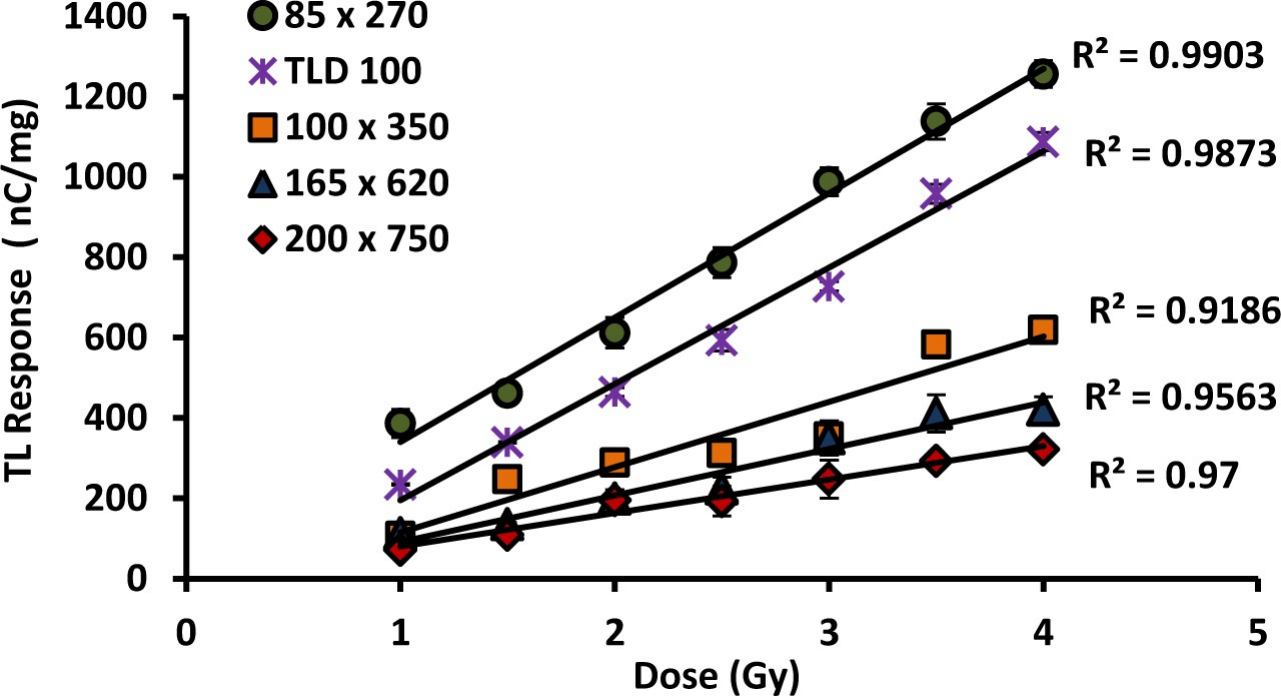
Dose response for different diameter of 6 mol% Ge–doped flat fibres in comparison to that of TLD-100 chips, provided together with the standard error of the mean.

**Fig 5:**
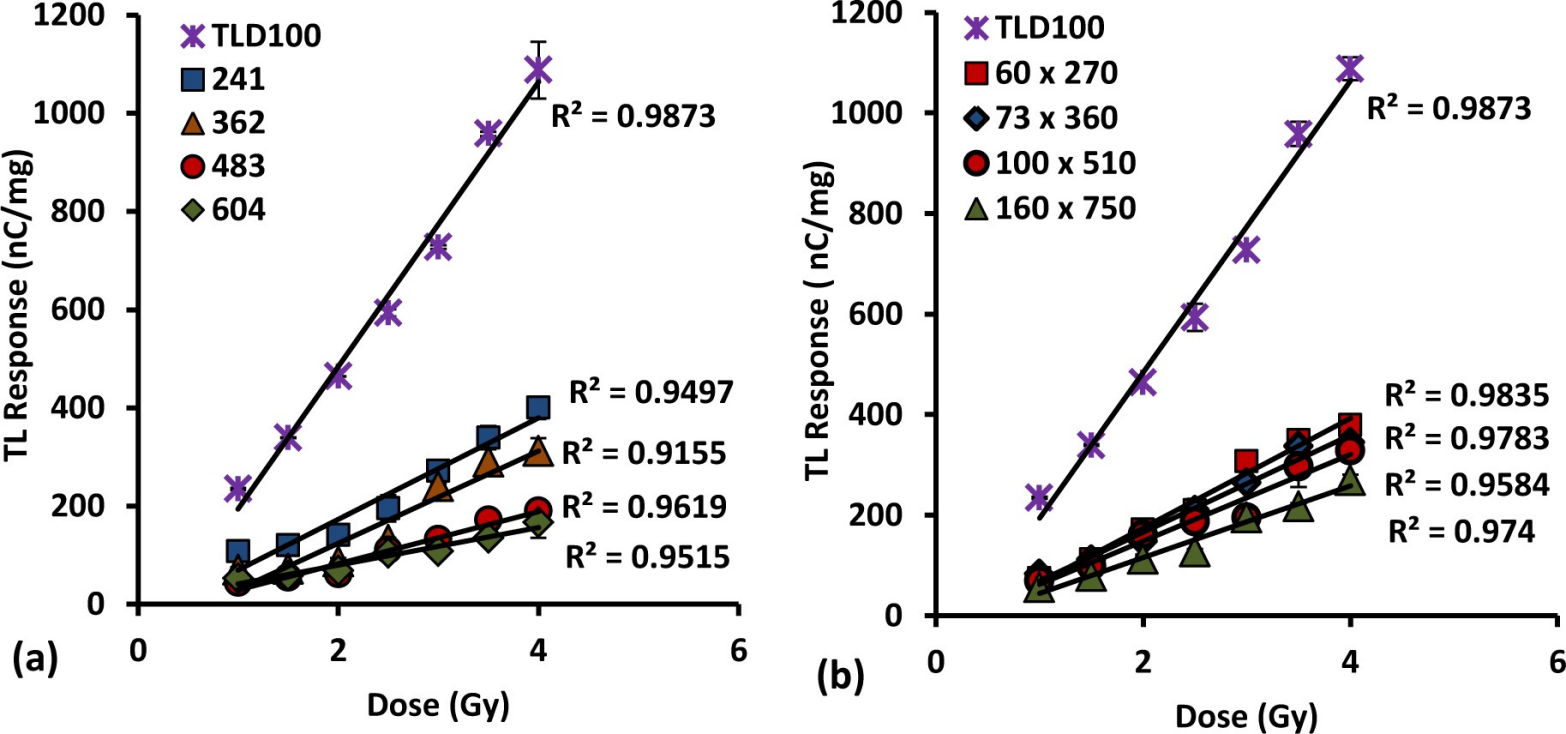
Dose response for different diameter of 8 mol% Ge–doped (a) cylindrical and (b) flat fibres in comparison to that of TLD-100 chips, provided together with the standard error of the mean.

The graphs of dose response for 6 and 8 mol % various dimension cylindrical and flat fibre cross-sections and the TLD-100 chips are shown in Figs 4 and 5. The results show linear for all cases entire the doses study and the linear TL response supporting findings from previous number of workers [16,27,30,40–41]. The increase in TL yield with dose remains linear over a wide range of values specifically from the 1Gy up to 4 Gy dose range investigated. Ideally, the linearity of responses have been measured across a much wider range of doses from previous work by several researchers and tabulated in Table 4. The results are important in that it is desirable for the measurement system to produce a linear response to absorbed dose in the range of doses given, according with the doses delivered in daily fractions of radiotherapy delivered to patients. The TLD-100 chips were used in order to validate the results obtained against the performance of a well-known TLD material.

**Table 4:** Linearity of responses have been measured across a much wider range of doses from previous work by several researchers.

Year	Researcher	Dosimetric Material	Dosimetric Characterisation	Electrons Irradiation (Dose (Gy) / Energy (MeV))
**2009**	Hashim et. al [26]	In comparison between commercial Ge-doped Al-doped optical fibres (INOCORP, Canada)	Dose Response and Fading	1–3.5 Gy (6, 9, 12 MeV)
**2011b**	Yaakob et. al [42]	In comparison between commercial Ge-doped Al-doped optical fibres (INOCORP, Canada)	Dose Response	0.02–0.24 Gy (9 MeV)
**2011**	Wagiran et. al [43]	Commercial Ge / Al doped optical fibres (INOCORP, Canada)	Dose Response	0.2–4 Gy (6MeV)
				In comparison to photon irradiation with 6 MV energy
**2013**	Alawiah et. al [15]	Tailor made undoped Flat Fibres	Dose Response, Glow Curve, Deconvolve Glow Curve, Energy Dependence and Sensitivity	2–10 Gy (6–21 MeV)
**2017**	Alawiah et. al [44]	Tailor made Germanium Flat Fibre and TLD-100	WinREMS glow curve analysis, WinGCF deconvoluted peak analysis, Supralinearity, TL sensitivity	1Gy -1Mgy (2.5 MeV)

Thus, generally speaking all of the fibres investigated herein are suitable for use as TLD materials. The dose response of 6 mol% Ge-doped cylindrical fibres has been reported elsewhere by Siti Nurasiah et. al (2016) [16]. For the flat fibres i.e., 200 x 750, 165 x 620, and 100 x 350 μm, these show TL yields of the order of 28%, 40% and 55% respectively of that of TLD-100 chips. For the smallest size of flat fibre (85 x 270 μm) a higher TL yield than that of TLD-100 is obtained, by a factor of 1.1. In comparing the TL response of the fibres, the flat fibre response is some 7.1 times that of the cylindrical fibres. This may be explained in terms of the dependence on surface area since the flat fibres have larger surface area than that of the cylindrical forms and as such more electron–hole traps are available for occupancy occurs in flat fibre forms.

Moreover, a comparison between the responses in TL yield per unit absorbed dose in terms of dimensions showed that the smallest dimension fibres of both types provide a response greater than that of the larger dimension fibres. For 8 mol% Ge-doped fibres, the 241 μm cylindrical fibre has the greatest TL response followed by 362, 483 and 604 μm cylindrical fibres, at of the order of 35%, 32%, 18% and 13% respectively of that of TLD-100 chips. Conversely, for the different dimension flat fibres i.e., 60 x 270, 73 x 360, 100 x 510 and 160 x 750 μm, it is observed that the TL yield in count per second per unit mass fibre is of the order of 38%, 33%, 30% and 25% respectively of that of to the TLD-100 chips. It is clear that the smallest dimension fibres are the most sensitive, decreasing with increasing dimension values. This condition has been discussed in detail in our previous study regarding the Beer-Lambert law [27]. To conclude, the TL dose response increases with reduction in fibre outer dimensions. Comparing the concentration of dopants in the fibres, the TL response of 6 mol% showed the greater response ~ 2.8 times that of the 8 mol% Ge-doped fibres, respect being made to response of the smallest dimension fibres.

All of the fibres tested herein satisfy the desirable themoluminescence characteristic of good linear response over the dose range. The flat fibres show the superior response. However in addition, the smallest dimension fibres of all types offer slightly greater dose response compared to the larger dimension fibres. The 85 x 270 μm 6 mol% Ge-doped flat fibre showed the overall best response among all other fibres tested herein, presenting the highest response even compared to TLD-100 chips. As a result that fibre can be considered to be preferable to use of TLD-100 chips where sensitive dose measurement capability is required. SiO_2_ optical fibres have become of interest among the available thermoluminescence materials for *in-vivo* studies in radiotherapy due to its small size and resistance to water [45]. From investigations on these fibres, it has been found that the response of fibre is influenced by the physical characteristic of the fibres, including their spatial resolution and the production of fibres through the MCVD process, introducing dopant at selected concentrations.

### 3.6. Linearity index, ƒ (D)

**Fig 6:**
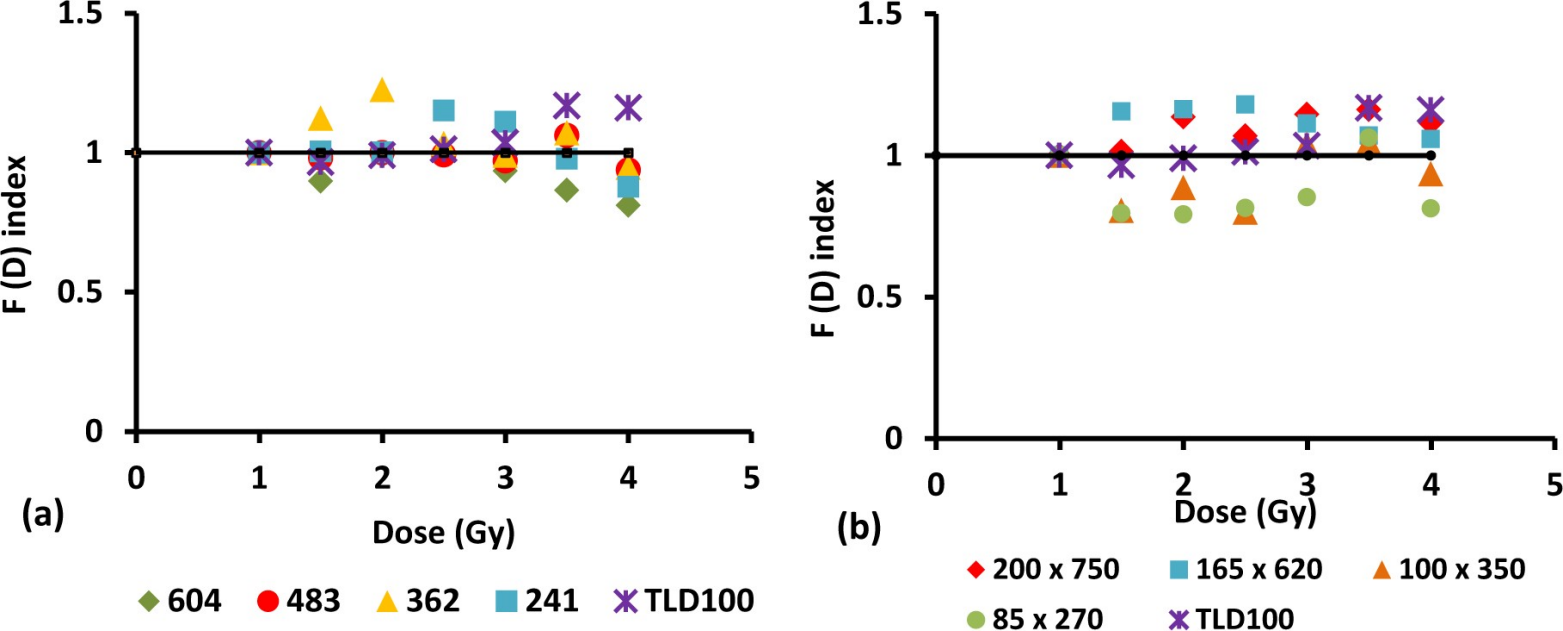
The linearity index versus dose for different diameter of 6 mol% Ge–doped (a) cylindrical and (b) flat fibres in comparison to that of TLD-100 chips subjected to electron irradiation.

**Fig 7:**
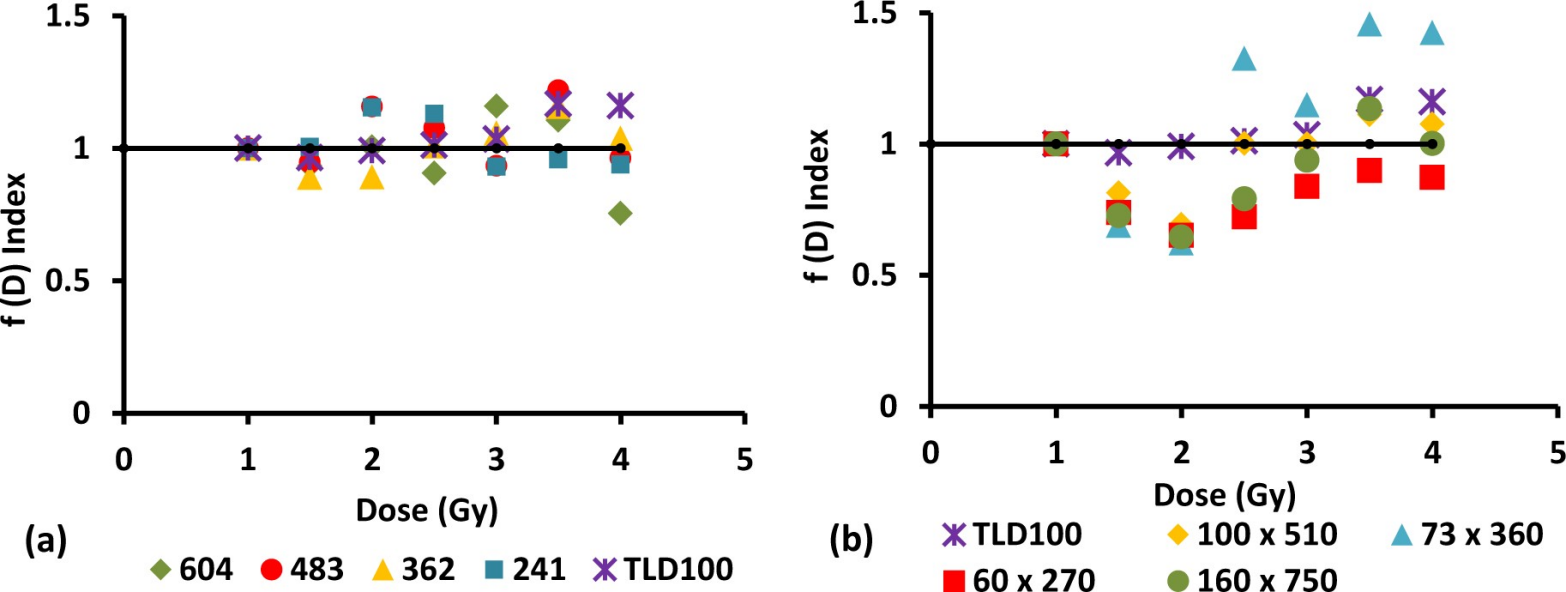
The linearity index versus dose for different diameter of 8 mol% Ge-doped (a) cylindrical and (b) flat fibres in comparison to that of TLD-100 chips subjected to electron irradiation.

The linearity index, ƒ(D), is of convenience in determining the normalized thermoluminescence (TL) dose response. The importance of the index lies in the fact that since an ideal dosimetric material should have a linear dose response over a wide dose range. However not all dosimetric materials that are used in practical dosimetry offer this, instead displaying a variety of non-linear effects. In particular, one often finds that the response of a TLD materials is linear, then supralinear and then sublinear as dose is increased [46]. This is because usually the supralinear response can be ascribed to large radiation doses causing damage to the medium and creating more traps thus increase filled traps while the sublinearity response is believed to occur when the TL material reaches saturation in electron-hole trap numbers. Other than that, this condition may be attributed to non–uniform spatial ionization density following electron irradiation. This phenomenon has been successfully introduced as the Unified Interaction Model (UNM) [39,47]. Therefore, to overcome this problem the linearity Index was determined using formula:

f(D)=M(D)DM(D1)D1 (2)

where M (D) is the intensity of TL signal at dose, M (D_1_) is the intensity of TL signal at low dose, D is the dose and D_1_ is the low dose. Therefore, when ƒ (D) = 1 linear behaviour is achieved, while for ƒ (D) > 1 the response is superlinear and when ƒ (D) < 1 it is said to be sublinear. The graph of ƒ (D) versus dose in Gy has been plotted to show the linearity of data for increasing dose. The linearity index versus dose for different dimension fibres is shown in Figs 6 and 7.

For diameter 604 μm of 6 mol% Ge- doped cylindrical fibres, the ƒ (D) were determined to be sublinear from 1.5 Gy to 4 Gy. For diameter 483 μm showed sublinear from 1.5 Gy to 4 Gy and became superlinear at 3.5 Gy, for diameter 362 μm; the result obtained superlinear from 1.5 Gy to 4 Gy except at 3 Gy became sublinear and for diameter 241 μm; the value of ƒ (D) is > 1 from 1.5 Gy to 3 Gy and < 1 from 3.5 Gy to 4 Gy. For the cross- section dimensions 200 x 750 μm and 165 x 620 μm of 6 mol% Ge- doped flat fibres, the value of ƒ (D) were found to be greater than one (superlinear). For the cross- section dimension 100 x 350 μm displayed sublinear from 1.5 Gy to 2.5 Gy and superlinear from 3 Gy to 4 Gy while for the smallest cross-section of flat fibre, i.e, 85 x 270 μm were found to be subliner from 1.5 Gy to 4 Gy except for 3.5 Gy where it is superlinear.

For diameter 604 μm and 483 μm of 8 mol% Ge-doped cylindrical fibre, the value of ƒ (D) show sublinear, superlinear and then back to sublinear within the range of dose delivered, 1 Gy to 4 Gy. For diameter 362 μm, the value of ƒ (D) is < 1 from 1.5 Gy to 2 Gy and > 1 from 2.5 Gy to 4 Gy while for diameter 241 μm is >1 from 1.5 Gy to 2.5 Gy and <1 from 3 Gy to 4 Gy. For the cross- section dimension 160 x 750 μm of 8 mol% Ge- doped flat fibres, the value of ƒ (D) were found to be less than one (sublinear) from 1.5 Gy to 3 Gy and to be greater than one from 3.5 Gy to 4 Gy. The value of ƒ (D) for the cross- section dimensions 100 x 510 μm and 73 x 360 μm show sublinear from 1.5 Gy to 2 Gy and superlinear from 2.5 Gy to 4 Gy. The variation in the linearity index for the flat fibres when compared to the cylindrical fibres may be due to effect from the sample itself, as in variation in the optical density, inhomogeneous distribution of dopant concentration along the core of the fibres, the differences in size and also the mass of the fibres [48]. The previous study by Abdul Sani using the PIXE/RBS method has been proved that the fibre fabrication process is typically seen to result in broad variation in dopant concentration and distribution, critical parameters in controlling the character of the dosimeter [49].

Apart from the physical properties that could affect the linearity of the fibres, the type and energy of ionizing radiation will also influence the linearity of fibres itself.

### 3.7. Sensitivity

**Fig 8:**
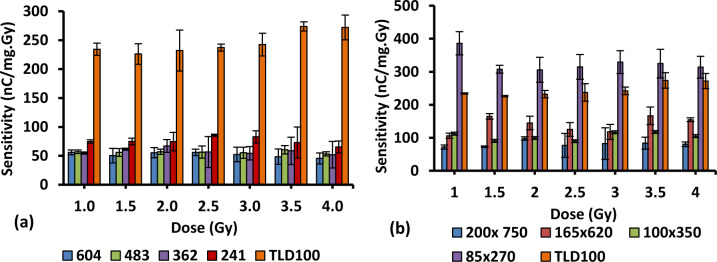
The sensitivities of 6 mol% Ge-doped (a) cylindrical and (b) flat silica fibre of different diameters and TLD-100 chips.

**Fig 9:**
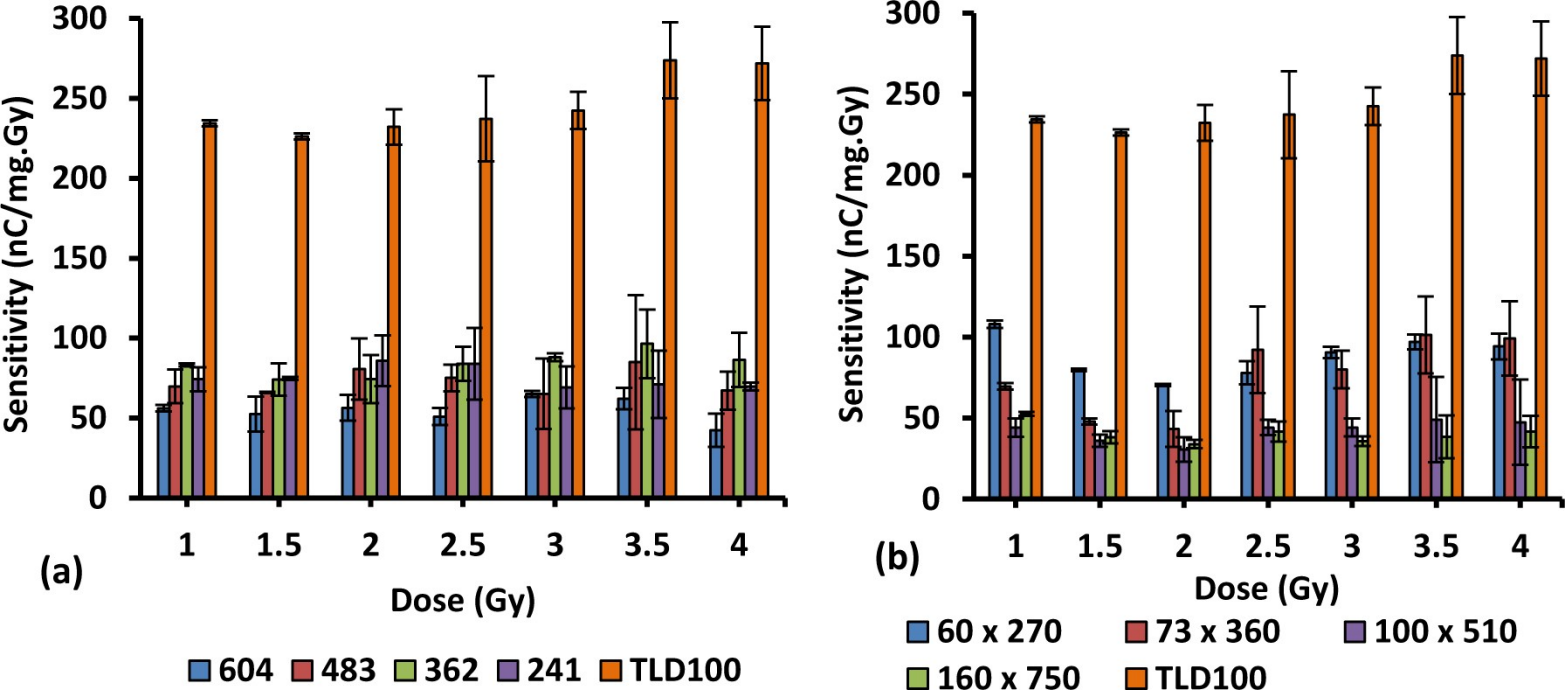
The sensitivities of 8 mol% Ge-doped (a) cylindrical and (b) flat silica fibre of different diameters and TLD-100 chips.

The sensitivity of different concentrations and diameters of Ge-doped optical fibres were calculated in units of nC.Gy^-1^.mg^-1^. The graph of sensitivity against dose for different dimension fibres has been plotted. Figs 8 and 9 provide the graphical summary of the TL sensitivity of the samples carried out in this work when subjected to 1 Gy to 4 Gy electron irradiation. For the 6 mol% Ge-doped fibres, the relative sensitivities were determined to be 0.21,0.23, 0.24 0.31,0.33, 0.57,0.43 and 1.33 for 604, 483, 362, 241, 200 x 750, 165 x 620, 100 x 350 and 85 x 270 μm fibres respectively with respect to TLD-100 chips as shown in Fig 9(A) and 9(B). For the 8 mol% Ge-doped optical fibres, the relative sensitivities were found to be 0.22, 0.29, 0.32, 0.33, 0.34, 0.31, 0.16 and 0.18 for 604, 483, 362, 241, 60 x 270, 73 x 360, 160 750 and 100 x 510 μm fibres respectively with respect to TLD-100 chips as presented in Fig 9(A) and 9(B). These results for the sensitivity of all the sample silica fibres are lower than sensitivity of TLD-100, the exception being results shown in Fig 8(B). Moreover, the smallest diameter for each type of fibre displayed the greatest sensitivity. Based on Fig 8(B), the results illustrate the sensitivity of the 85 x 270 μm fibres to be superior to that of the larger diameter fibres and also of the sensitivity of TLD-100. Thus it is also important to observe that the greatest spatial resolution fibre, with dimension 85 x 270 μm, provided the greatest sensitivity, being suitable for medical applications at the applied doses.

From the graphs, it can further be seen that values of sensitivities are not arranged in order from smaller to larger size. The variation in sensitivity between fibres may be a result of inhomogeneous dopant concentration, varying from point to point along the length of the fibres, non-uniformities in distribution of added dopant in the core influencing the TL response distribution of the optical fibres. Moreover, a comparison between the average value of sensitivity fibres with respect to TLD-100 for 6 and 8 mol% Ge-doped fibre show 6 mol% fibres to be ~ 1.5 times greater than that of 8 mol% Ge-doped. It can be concluded that the sensitivity of the optical fibres are dependent on the cross-sectional dimensions of the optical fibres and the concentration of Ge-dopant in the fibres. The difference in sensitivity at the higher dose is due to low optical signal at these doses, giving a high signal-to-noise ratio [50].

### 3.8. Energy dependence

**Fig 10:**
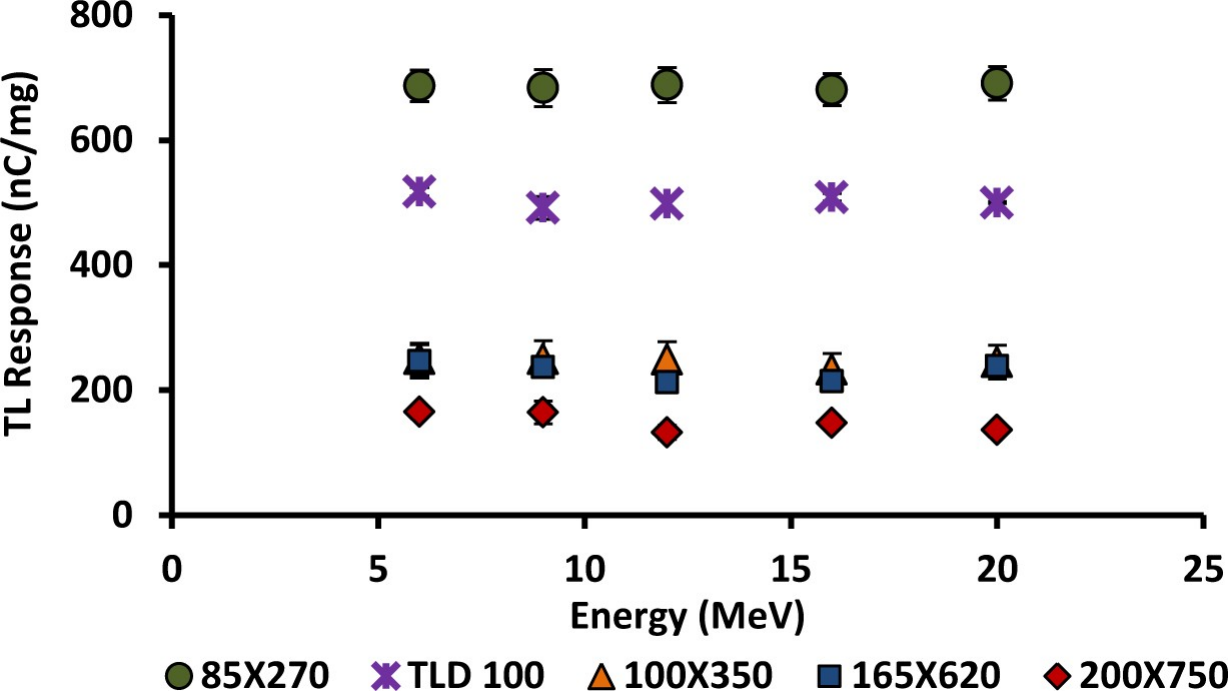
TL response for 6 to 20 MeV electron beams for the 6 mol % Ge-doped flat fibres and TLD-100 chips, exposed to 2 Gy of dose at 400 MUmin^-1^.

**Fig 11:**
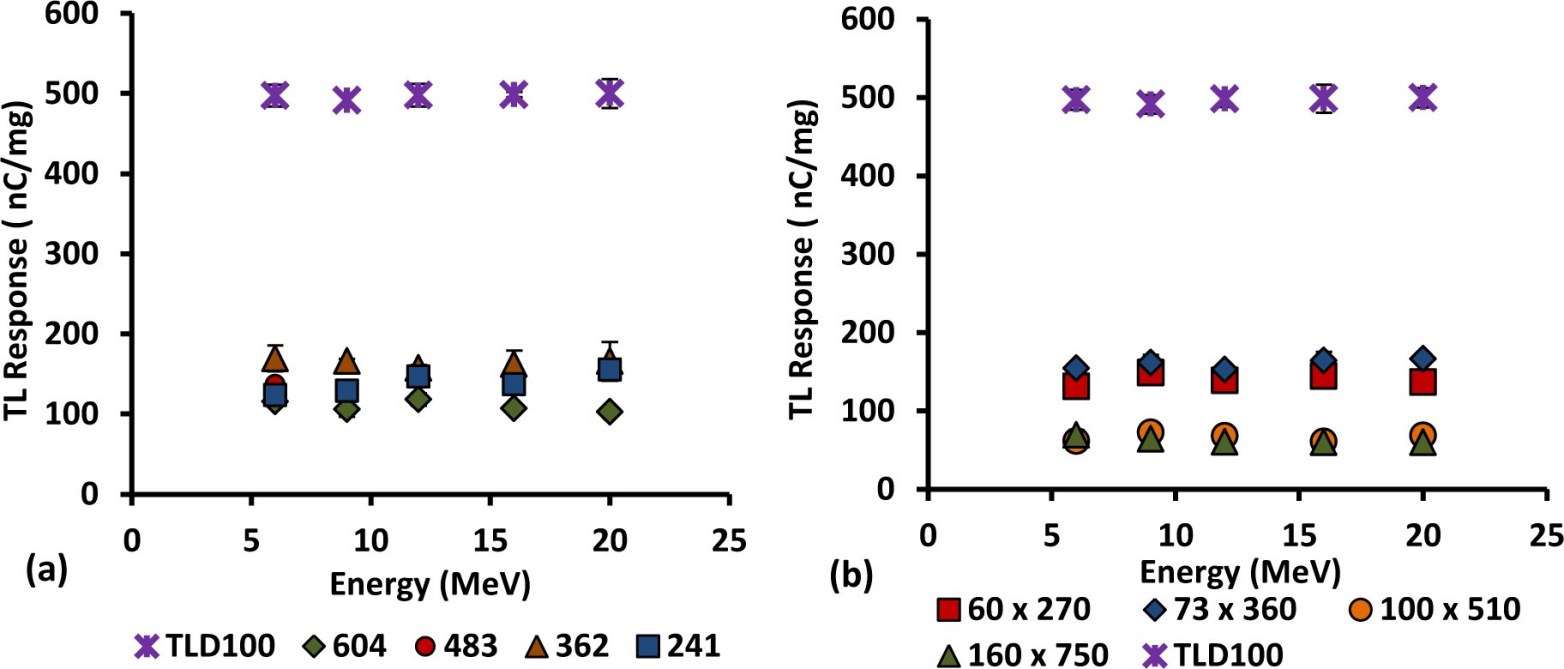
TL response for 6 to 20 MeV electron beams for the 8 mol % Ge-doped (a) cylindrical and (b) flat fibres of various diameters and TLD-100 chips, exposed to 2 Gy of dose at 400 MUmin^-1^.

The energy dependence of 6 and 8 mol% Ge-doped cylindrical and flat fibres of different cross-sectional dimensions were investigated together with TLD-100 chips. All fibre samples and TLD-100 chips were irradiated using various energy electron beams as mentioned, with a range of nominal energies as used in radiotherapy, namely 6, 9, 12, 16 and 20 MeV, exposed to an absolute dose of 2 Gy at a dose rate of 400 MUmin^-1^. The graph of TL yield versus Energy (MeV) is presented in Figs 10 and 11(A) and 11(B). Each data point was obtained by taking the mean of five individual fibre readings, normalised by mass. All the results show no significant change in TL signal output with increase in energy, the energy response being essentially the same within the range of energies delivered 6–20 MeV. Independence of energy of 6 mol% cylindrical fibres has also been reported in previous study [16,15,51]. The requirement of an ideal TL material is thus satisfied, that it be tissue equivalent and energy independent, allowing one to obtain the same radiation properties as the tissue at all possible energies [52].

### 3.9. Dose rate dependence

The irradiations were made a fixed dose of 2 Gy at a constant electron beam of nominal energy 6 MeV and delivered at dose-rates in the range 100 to 600 MUmin^-1^. A bolus material was used as build-up in this case. The samples were placed at depth d_max_ for each measurement using a field size of 10 x 10 cm^2^ and a source–to–surface distance (SSD) of 100 cm. Each data point was obtained as the mean of five sample readings and the results were normalised by the mass of each individual fibre. The error bars were calculated from the standard deviation of the readout of the five samples readings for each diameter of fibre.

**Fig 12:**
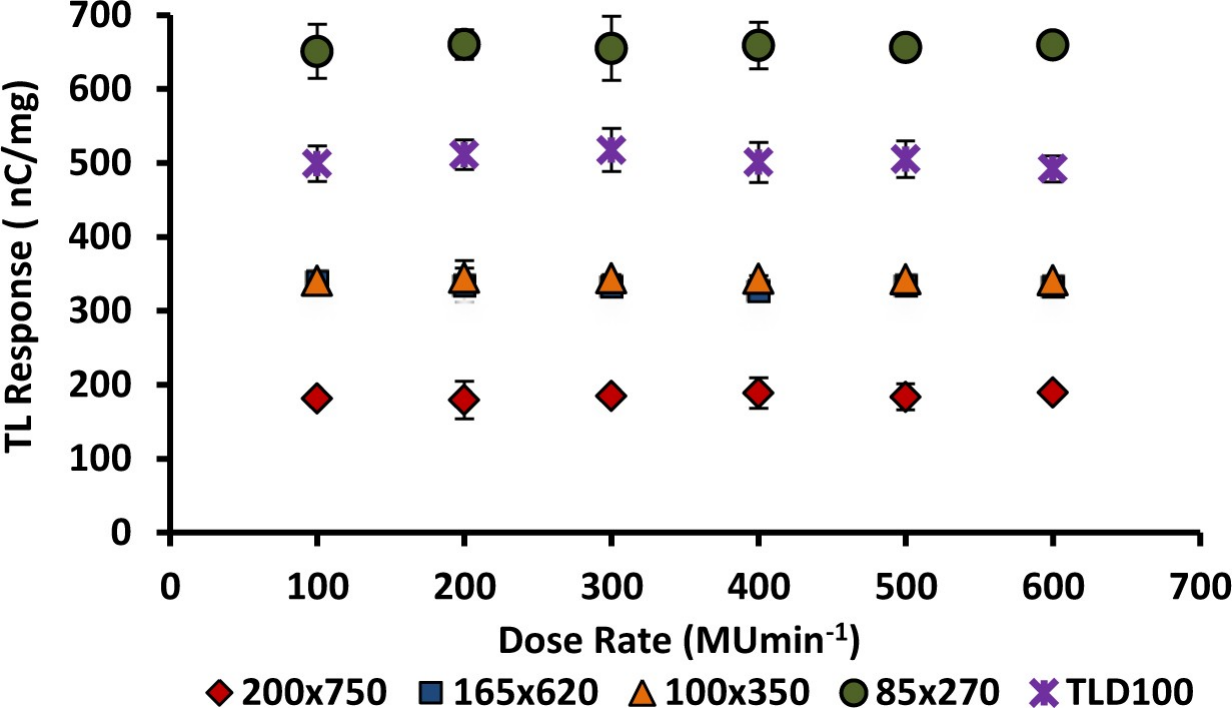
TL response for 6 MeV electron beam delivered at dose-rates in the range 100 to 600 MU min^-1^ for the 6 mol % Ge- doped flat optical fibres and TLD-100 chips, exposed to a dose of 2 Gy.

**Fig 13:**
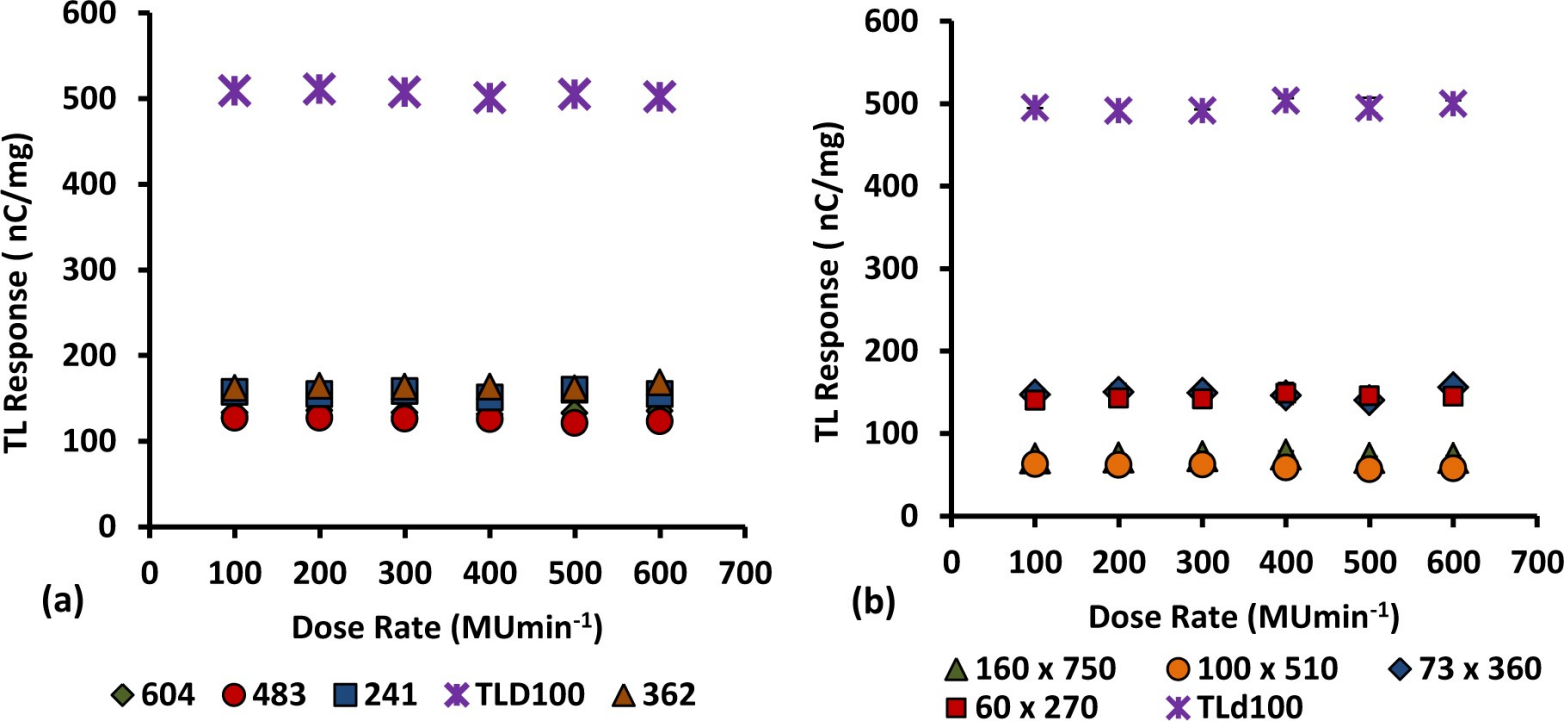
TL response for 6 MeV electron beam delivering in the dose-rate range 100 to 600 MU min^-1^ for the 8 mol % Ge-doped (a) cylindrical and (b) flat fibres with different cross-sectional diameters and TLD-100 chips, exposed to a dose of 2 Gy.

Figs 12 and 13(A) and 13(B) show the effect of dose-rate on the TL yield of cylindrical and flat fibres of various dimensions, with Ge dopant concentrations of 6 and 8 mol%, together with TLD-100 chips. The dose rate dependence of 6 mol% Ge-doped cylindrical fibres was reported previously [16]. The graphs clearly show there to be no significant change in TL yield from lower to higher dose-rate. Thus, the results show the TL response of fibres and TLD100 chips to be independent of dose-rate within the range 100 to 600 MUmin^-1^. The finding is supported by previous study [35,52] have reported specifically for TL LiF phosphors (e.g. as used in Harshaw TLD-100) that to be a useful themoluminescence dosimeter medium it should be independent of dose-rate (up to 10^9^ Gy s^-1^). All diameters of 6 and 8 mol % Ge-doped silica optical fibres tested herein are suitable for use as a clinical dosimeter without any dose-rate calibration since all of them are independent of dose rate. This characteristic is vitally important in medical application.

### 3.10. Reproducibility

**Fig 14:**
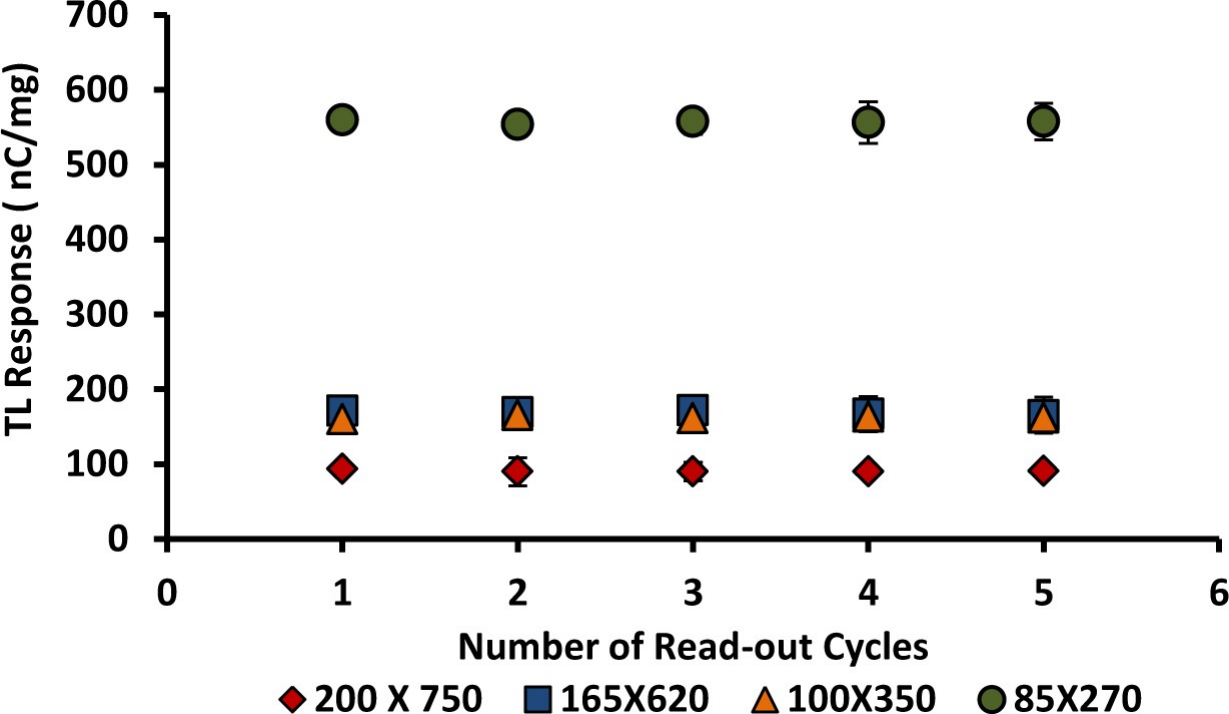
Reproducibility for the various cross-sectional dimensional 6 mol% Ge-doped flat fibres exposed to electron doses of 2 Gy, shown together with the standard error of the mean.

**Fig 15:**
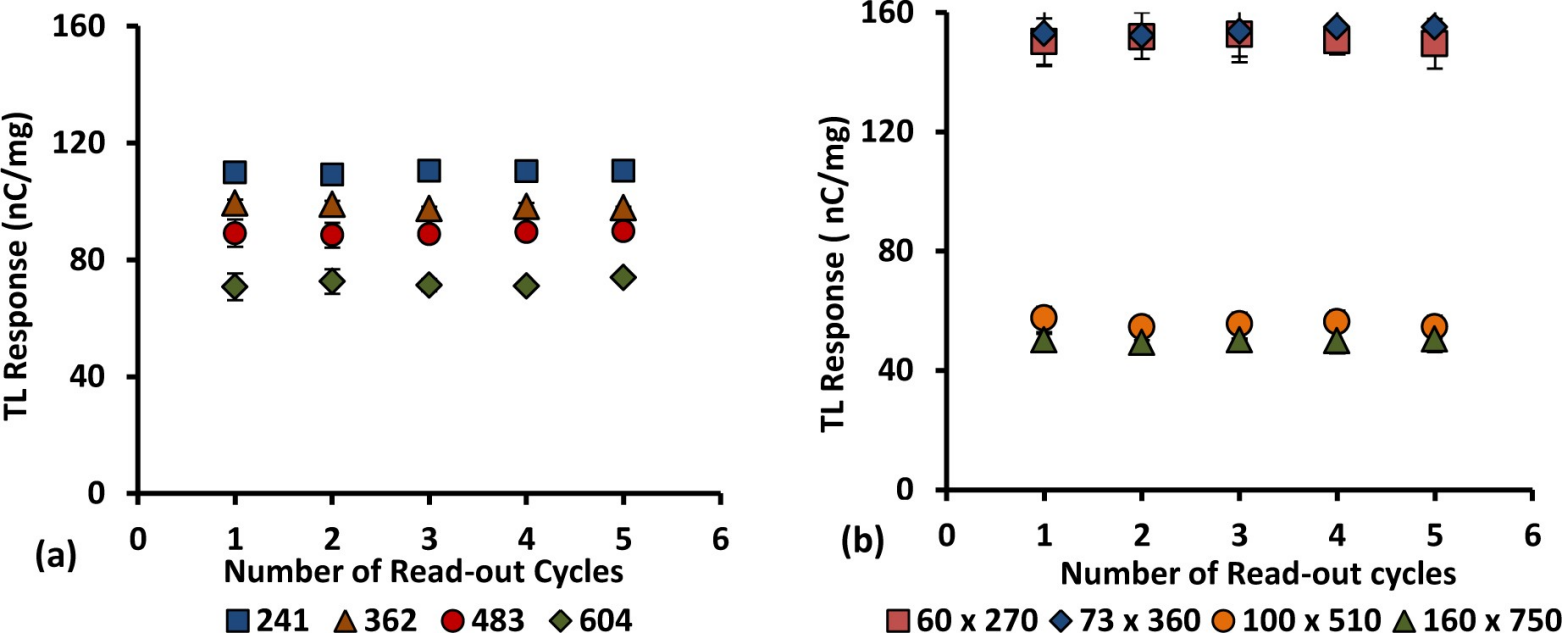
Reproducibility for the various cross-sectional diameter of 8 mol% Ge-doped (a) cylindrical and (b) flat fibres, exposed to electron doses of 2 Gy, shown together with the standard error of the mean. (Note: in some instances the error bars are smaller than the size of the individual data points).

Three samples of each form of 6 and 8 mol % Ge-doped cylindrical and flat fibres were exposed to a constant dose-rate of 400 MUmin^-1^ using a clinical electron beam operated at nominal energy 6 MeV. The samples were irradiated to a dose of 2 Gy using a standard source-to-surface distance (SSD) of 100 cm and a field size of 10 x 10 cm^2^ in a series of five repeat cycles of irradiation, measurement and annealing. The graph of TL yield per unit mass of the fibres against the number of repeat cycles clearly shows the ability for the samples to be re-used multiple times. Based on the results presented in Figs 14 and 15, no significant differences are found between the five repeat measurements for all of the samples studied. All of them show good reproducibility with a standard deviation of < 2% and < 4% for 6 and 8 mol% Ge-doped fibres respectively. The 6 mol% Ge-doped fibres show somewhat better results compared with the 8 mol% Ge–doped fibres. However, in general < 5% reproducibility is acceptable in medical dosimetry [42]. Herein, the results clearly show all the samples to be suitable for use as TL dosimeters since all of them are able to produce a TL response that is effectively the same after reuse without any noticeable change in TL response.

### 3.11. Fading

**Fig 16:**
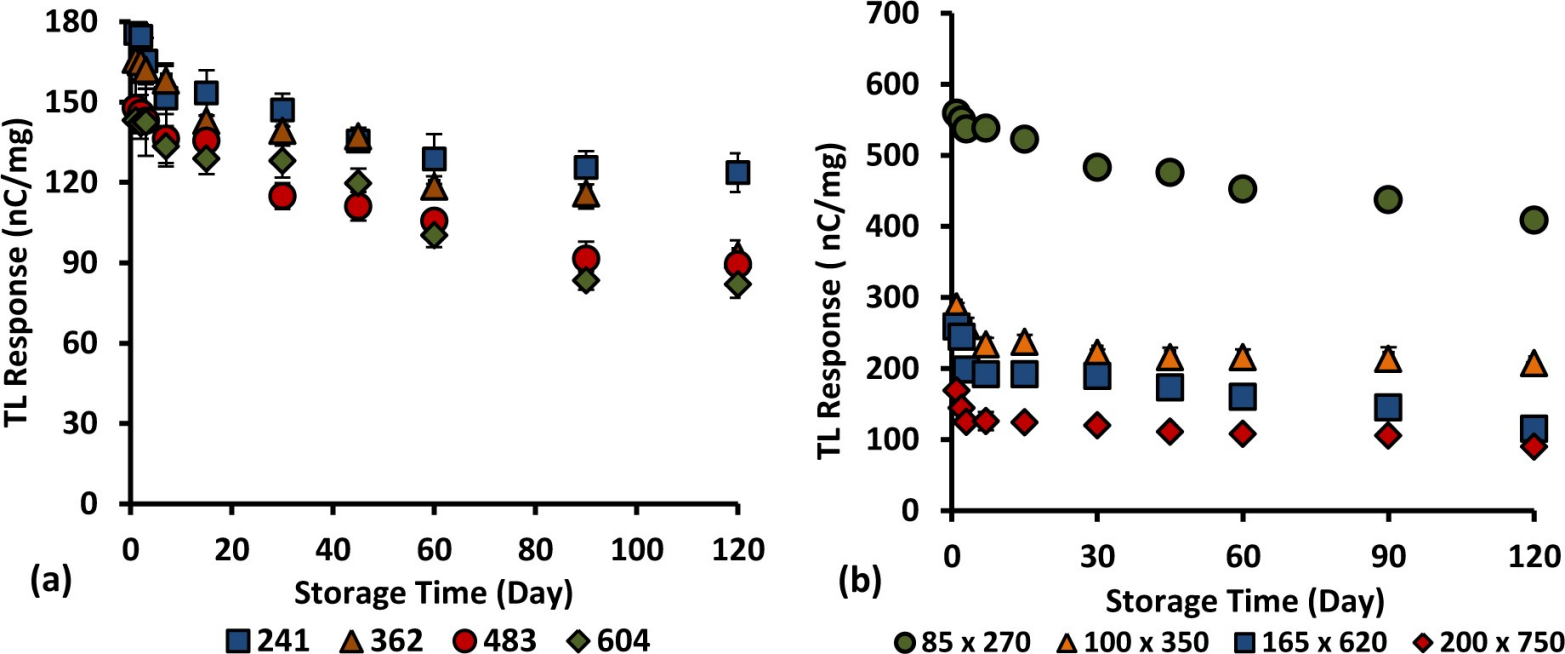
The loss of TL yield over a period of 120 days post- irradiation for 6% mol Ge-doped (a) cylindrical and (b) flat optical fibre irradiated to 2 Gy of electron beams.

**Fig 17:**
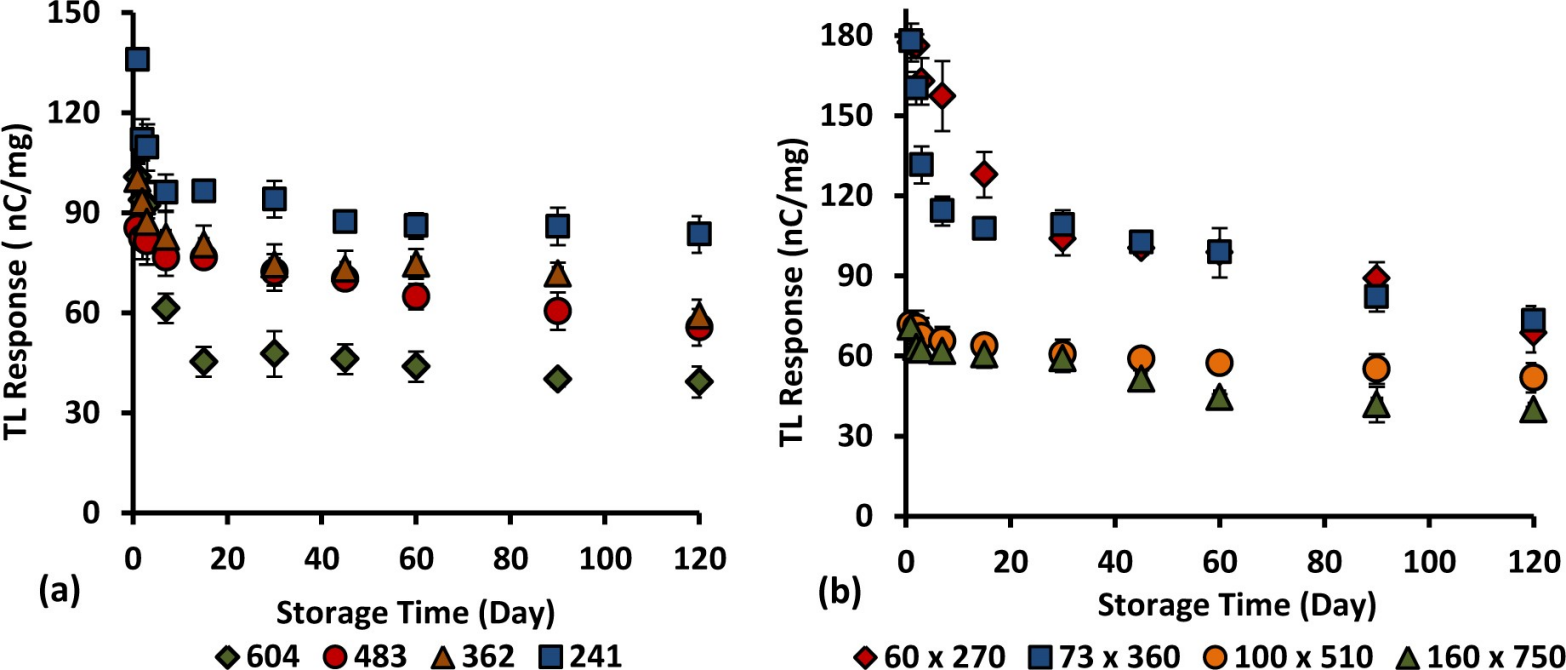
Loss of TL yield over a 120 day period post-irradiation for 8 mol % Ge-doped (a) cylindrical and (b) flat fibre irradiated to an electron dose of 2 Gy.

Herein, the analysis of fading effect of 6 and 8 mol% Ge-doped fibres of different cross-sectional dimensions and types was investigated over a period of 4 months in order to investigate the effect of absorbed dose on the signal stability. The fibre samples were irradiated simultaneously to a dose of 2 Gy at 400 MUmin^-1^ using 6 MeV and subsequently kept in the dark at a room temperature. The graphs of TL yields (counts per second per unit mass of fibre) against storage time (days) have been plotted, as shown in Figs 16(A) and 16(B) and 17(A) and 17(B). Based on the graphs, all the fibres show the TL yield to decrease over a period of 120 days of storage time. For the 6 mol% Ge–doped fibres, the loss in TL yield was found to be less than 50% over 120 days post-irradiation, one exception being for the flat fibre of cross-sectional dimensions 100 x 350 μm (showing a 55.4% loss). For the cylindrical and flat 6 mol% Ge–doped fibres, over the period studied both of them displayed the lowest signal loss for the smallest dimension fibres, i.e., 241 μm and 85 x 270 μm, showing loss in TL yields of around 29.7% and 26.9% respectively. For cylindrical fibres, the average loss in TL yield was estimated to be 0.25% - 0.37% per day within the first 4 month period post-irradiation while for flat fibres the signal loss was estimated to be 0.22% - 0.46% per day within 120 days post-irradiation.

For the 8 mol% Ge–doped fibres, the loss in TL yields were found to be less than 50% over 120 days post-irradiation, specifically for fibres of cross-sectional dimensions 483, 362, 241, 160 x 750 and 100 x 510 μm, while it was greater than 50% over 120 days post-irradiation for fibres of cross-sectional dimensions 604, 73 x 360 and 60 x 270 μm. After 4 months, the largest signal loss was 61.3%, suffered by the 60 x 270 μm flat fibres while the smallest signal loss was 27.8%, contributed by the 100 x 510 μm fibre. For cylindrical fibres, the average loss in TL yield was estimated to be 0.29% - 0.51% per day within 4 months of irradiation while for flat fibres the signal loss was estimated to be 0.23% - 0.51% per day within 120 days of irradiation. Present investigation reveals the 85 x 270 μm flat fibre to have the most superior performance, offering the least loss in TL yield within 120 days post-irradiation.

## 4. Conclusion

Present work demonstrates that the proposed dosimeter fibres of different cross-sectional dimensions and dopant concentrations to be suitable for use as TL dosimeters, with reusability without any particular regeneration treatment, dose rate and energy independence and good linearity of response. Apart from this, the TL response of Ge-doped optical fibres clearly shows them to be capable of providing precise and accurate dosimetric evaluation for doses in areas subjected to acute radiation dose delivery.

Further to the above, the flat fibres have excellent response and high dose sensitivity, greater than that of the cylindrical fibres for both dopants (6 and 8 mol%). From these results, the proposed flat optical fibres can be used as radiation sensors for dosimetry and favourably compare with the cylindrical optical fibres. Therefore, these flat fibres shows several promising dosimetric features to be introduced as a new TL material in place of the TLD-100 (LiF:MgTi) dosimeter. The 85 x 270 μm flat fibre with 6 mol % Ge-dopant concentration showed the greatest response among all of the fibres and also TLD-100 chips. Definitely, it is apparent that there is a flourishing improvement in TL response, scaling as an inverse function of fibre cross-section dimensions. Generally, the 6 mol% Ge-doped flat optical fibres have been shown to possess highly desirable characteristics as a sensor for measuring absorbed doses during radiotherapy treatments compared with 8 mol%. Based on the response of the smallest diameter for both flat optical fibre the percentage coefficient between 6 mol% and 8 mol% is about 72%. It is expected that the TL output increases with dopant concentration since the number of electron traps and recombination centres increases with dopant concentration. When the concentration of dopants increased, the TL response of the samples decreased. This happens because the distances between traps become shorter as the number of traps and recombination centres increases, raising the probability for the light emitted from one recombination process to be absorbed by an electron in another trap, a phenomenon called self absorption [53]. Therefore, based on the collection of results, indicating that comprehensively the TL characteristics for 6 mol% Ge-doped optical fibres offer superior response and the several key properties of this TL fibre media is typically close to that of the TLD-100 chips.
