# Correction: Tailored Ge-doped fibres for passive electron radiotherapy dosimetry

**DOI:** 10.1371/journal.pone.0258264

**Published:** 2021-09-30

**Authors:** Siti Nurasiah Mat Nawi, S. F. Abdul Sani, M. U. Khandaker, N. M. Ung, K. S. Almugren, F. H. Alkallas, D. A. Bradley

In the Funding statement, the name of the funder is spelled incorrectly. The correct spelling is Deanship of Scientific Research at Princess Nourah bint AbdulRahman University, Riyadh.
